# Connecting the dots: spots on the skin, weakness within

**DOI:** 10.1007/s15010-024-02227-8

**Published:** 2024-03-13

**Authors:** Luisa Freyer, John Michael Hoppe, Inas Saleh, Stefan Brunner, Judith Spiro, Julius Steffen, Eleni Pappa

**Affiliations:** 1 Department of Medicine I, LMU University Hospital, LMU Munich, Munich, Germany; 2 Department of Medicine IV, LMU University Hospital, LMU Munich, Munich, Germany; 3 Department of Radiology, LMU University Hospital, LMU Munich, Munich, Germany

**Fig. 1:**
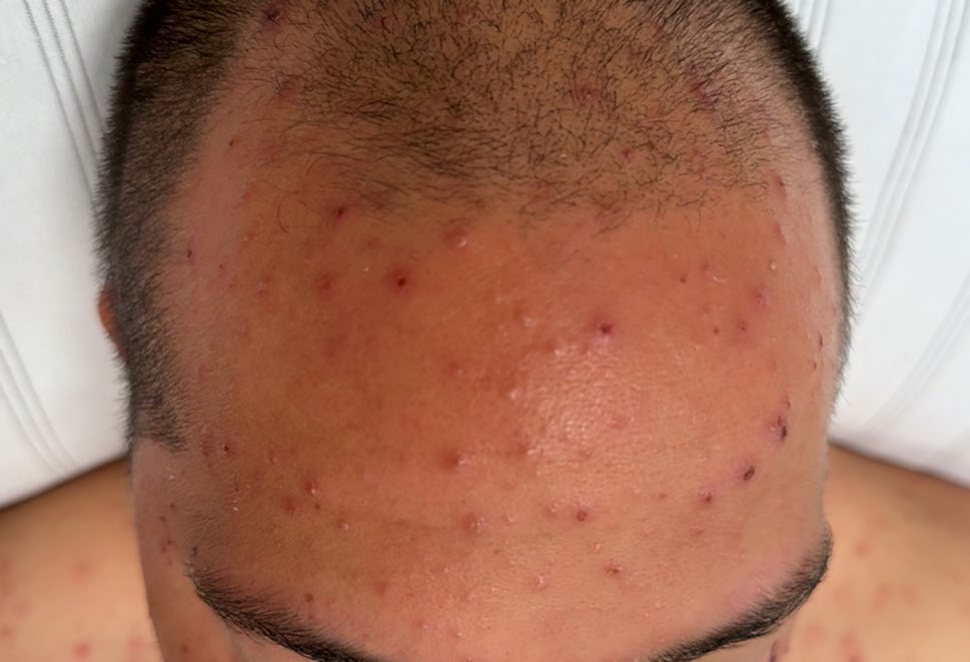
Photograph of the patient’s forehead with typical findings of chickenpox

A 36-year-old man presented to the emergency department after syncope accompanied by chest pain. He had a subfebrile temperature (38 °C) two days prior to admission, and his only significant medical history was a thyroidectomy for Graves’ disease seven years ago. On examination, he was hemodynamically stable, with a notable itchy rash of partially scarred vesicles and papules on his chest and face (Fig. 1). Within a few days, the rash had quickly spread to cover most of his body.

Laboratory findings revealed an elevated C-reactive protein (4.2 mg/dl) and leukocytosis (10.5 G/l), as well as elevated high-sensitive troponin T (0.041 ng/ml), creatine kinase (1606 U/l), N-type pro-brain natriuretic peptide (NT-proBNP) (525 pg/ml), and D-dimers (2.9 µg/ml). ECG showed a normal sinus rhythm, and echocardiography findings were inconspicuous. Pulmonary embolism and aortic dissection were ruled out by chest contrast-enhanced computed tomography.

**Fig. 2:**
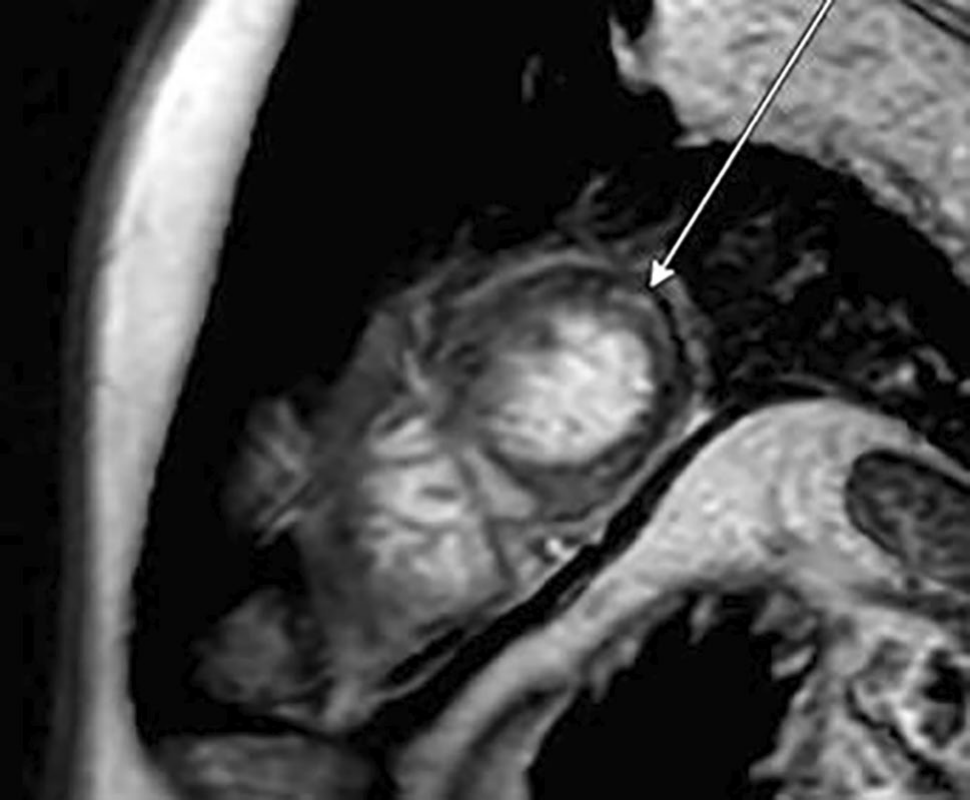
Cardiac magnetic resonance imaging (MRI). Phase-Sensitive Inversion Recovery Turbo Field Echo (PSIR TFE) sequences in short axis view show midmyocardial late gadolinium enhancement in the apical lateral wall of the left ventricle (arrow)

The following day, troponin levels surged 20-fold to 0.807 pg/ml. With coronary angiography revealing no abnormalities, myocarditis was suspected. Cardiac magnetic resonance imaging (MRI) revealed global edema and late gadolinium enhancement in the apicolateral wall of the left ventricle, typical for acute myocarditis (Fig. 2) [1].

As the dermatologic findings were suspicious for chickenpox and cardiac involvement was suspected, intravenous acyclovir (500 mg thrice daily) was initiated. Subsequently, inflammatory and cardiac markers normalised within seven days.

The patient had no history of chickenpox or shingles, was varicella zoster virus (VZV)-seronegative, and not immunocompromised. Vesicular fluid polymerase chain reaction confirmed primary VZV infection.

While chickenpox is uncommon in adults due to immunization schedules and childhood exposure [2], it should be considered a differential diagnosis for adults presenting with an unusual rash. If a primary VZV infection is suspected, patients should be evaluated for possible severe complications, such as encephalitis, hepatitis, pneumonia, or myocarditis, since mortality rates are dramatically higher in adults compared to children [3, 4].
